# The polychromatism of postmortem cerebrospinal fluid

**DOI:** 10.1007/s12024-024-00887-4

**Published:** 2024-08-30

**Authors:** S. Trella, C. Reinert, H. Heinsen, U. Preiß, CM. Monoranu, J. Zwirner, B. Ondruschka, M. Bohnert, S. Bohnert

**Affiliations:** 1 Institute of Forensic Medicine, University of Wuerzburg, Versbacher Str. 3, 97078 Wuerzburg, Germany; 2 Department of Neuropathology, Institute of Pathology, University of Wuerzburg, Josef-Schneider-Str. 2, 97080 Wuerzburg, Germany; 3 Institute of Legal Medicine, University Medical Center Hamburg-Eppendorf, Butenfeld 34, 22529 Hamburg, Germany; 4 Department of Oral Sciences, University of Otago, 310 Great King Street, Dunedin, 9016 New Zealand

**Keywords:** Forensic neuropathology, Forensic neurotraumatology, Biomarker, Immunocytochemistry, Cytology

## Abstract

Based on the assumption that postmortem cerebrospinal fluid (CSF) is contaminated depending on the chosen sampling technique in the forensic setting resulting in bloody or at least hemolytic CSF samples, we systematically documented a total of 183 postmortem CSF samples. These samples were all assessed for their quality and color, regardless of the cause of death or the postmortem interval. The investigations were carried out through subjective assessment of color and turbidity, as well as objective measurements of the optical density (OD) of the CSF supernatants after centrifugation of each sample, with standardized photographic documentation. The observations revealed that in 28 cases the CSF was absolutely (crystal-) clear and transparent. Most of our samples showed color changes ranging from xanthrochromic to rose. Intensive staining of the supernatants was only found in a small proportion of the examined collective. We found that postmortem CSF has no uniform appearance but rather a diverse range of color spectra, and the color, as well as the OD of the CSF, correlates significantly with the postmortem interval (p < 0.001) when sampled using the proposed standard procedure.

## Introduction

In clinical diagnostics, cerebrospinal fluid (CSF) is frequently sampled as a test substrate [1], as it communicates with the extracellular space of the central nervous system (CNS) without any barriers, and its analysis complements neuropathological investigations. Due to its protected anatomical location inside the skull and spinal canal [2], CSF is a promising test substrate. It is described as relatively stable, with only minor changes in the early postmortem phase [3], and its collection by suboccipital puncture or sampling following removal of the brain can be easily integrated into the autopsy routine. In the forensic field, CSF has been used, for example, for biochemical investigations of the postmortem interval (PMI) [4–7] and the application of different CNS biomarkers as an additional tool in forensic neuropathological diagnostics (“neuroforensomics”), such as after head injuries [8–11]. Furthermore, a recent study demonstrated that the CSF density of TMEM119-positive microglial cell profiles can also be used as a surrogate marker for neuropathological processes involving the CNS [12]. Since then, CSF analysis has become a promising diagnostic tool in interpretating the circumstances of death, although the biochemical analysis of CSF has not yet become routine in legal medicine, except in specific cases or investigations.

A recurring problem in the examination of postmortem CSF is contamination, particularly with blood admixture, depending on the chosen sampling technique in the forensic setting. This often results in bloody or at least hemolytic CSF samples, which may lead to limited assessment and reliability of the sampled material [13, 14]. Such artificial and iatrogenic blood admixtures also alter the color and visual appearance of postmortem CSF.

In the present study, we analyzed the visual aspects and performed subsequent photometric measurements of 183 postmortem CSF supernatant samples, independent of different causes of death and postmortem intervals (PMI). The objective of our study was to illustrate the immense *diversity* of postmortem CSF colors under the following hypothesis: the color of postmortem CSF correlates with PMI across different types of fatalities.

## Materials and methods

### Sampling and processing

A total of 183 CSF samples were collected as part of routine autopsies in 2021. Additionally, for each of the included cases, data were recorded on sex, age at death, and PMI, based on information from the death certificates as well as the final cause of death (determined after autopsy or through further toxicological, biochemical and / or histological analyses). Individual causes of death (CoD) were categorized into the following superordinate categories: cardiac death, multiple organ failure (MOV), hypoxia, intoxication, exsanguination, traumatic brain injury (TBI), isolated thoracic trauma (ITT), and undeterminable. There was no selection of specific deaths or exclusion of individual cases, which allowed for a strict consecutive sampling. All consecutive cases were included if it was possible to preserve at least a minimum amount of 1000 µl of CSF during autopsy. CSF was collected after opening the skull and before the removal of the brain from the cranial cavity, by puncturing the spinal canal through the foramen magnum with a non-sterile disposable pipette. The CSF samples were then transferred to polystyrene round-bottomed reagent tubes (A. Hartenstein GmbH, Wuerzburg, Germany). The individual CSF samples were immediately cooled at 4 °C and subsequently centrifuged at 5000 rpm for 5 min (Hettich Centrifuge Rotina 35, Andreas Hettich GmbH & Co.KG, Tuttlingen, Germany) to reduce the effects of potential contamination with blood cells. Centrifugation resulted in the separation of the CSF into a precipitate, with a cell pellet at the bottom of the test tube, and a liquid supernatant. 750 µl of the supernatant was carefully transferred to a fresh test tube.

### Visual assessment and measurements

The test tube was then documented photographically under standardized conditions using artificial light with equal exposure and illumination (iPhone XS, Apple Inc., Cupertino, California, USA) against a neutral gray background [15]. An overview image and a more detailed picture of the preserved supernatant were taken for all CSF samples. Additionally, the subjective color and turbidity of the CSF supernatant were assessed and documented according to the four-eyes principle per consensus and by lack of knowledge of PMI of each cadaver. The color was categorized subjectively into seven distinct categories (colorless, xanthochromic, yellow, rose, light red, red, dark red) after evaluating and reviewing all samples according to the four-eyes principle.

To objectively determine the individual color of each CSF sample, a central section of the supernatant image was selected. These image sections were then used to define the additive color code for each individual CSF supernatant using the RGB color model (red-green-blue) with *Microsoft Paint Software* (Microsoft Corporation, Redmond, Washington, USA) and the open-source image processing package *Fiji*. Care was taken to avoid artifacts caused by exposure-related reflections in the test tube. The raw data, including the red, blue, and green components of the color values obtained, were documented for each case. A simple yet specific color tile was created for each individual case using color coding to visualize and illustrate the color spectra for visual comparison.

Furthermore, the supernatant samples were analyzed for their individual optical density (OD). To measure OD, 200 µl of each supernatant was transferred into sterile, protein-free single sealed cuvettes (UVette, 220–1600 nm; Eppendorf SE, Hamburg, Germany). Before measuring the CSF supernatants, an individual blank value was determined by measuring highly purified water (Water HPLC grade; PanReac AppliChem, ITW Reagents Division, Darmstadt, Germany) using identical cuvettes in all test series. Measurements for both water and CSF were performed at a wavelength of 600 nm using an Eppendorf BioPhotometer (OD600; Model 6131, Eppendorf SE; Hamburg, Germany).

### Statistical analysis

To detect significant correlations between CSF coloration and the other measured values and characteristics, the data were analyzed using the Spearman correlation test in *IBM SPSS Statistics V27.0* (IBM Corp., Armonk, US). To account for multiple testing and control the false discovery rate (FDR), the Spearman test results were adjusted using the Benjamini-Hochberg correction. In this study, only *p*-values that remained statistically significant after correction (p(adj) < 0.05) are presented and discussed for clearer visualization.

## Results

**Fig. 1:**
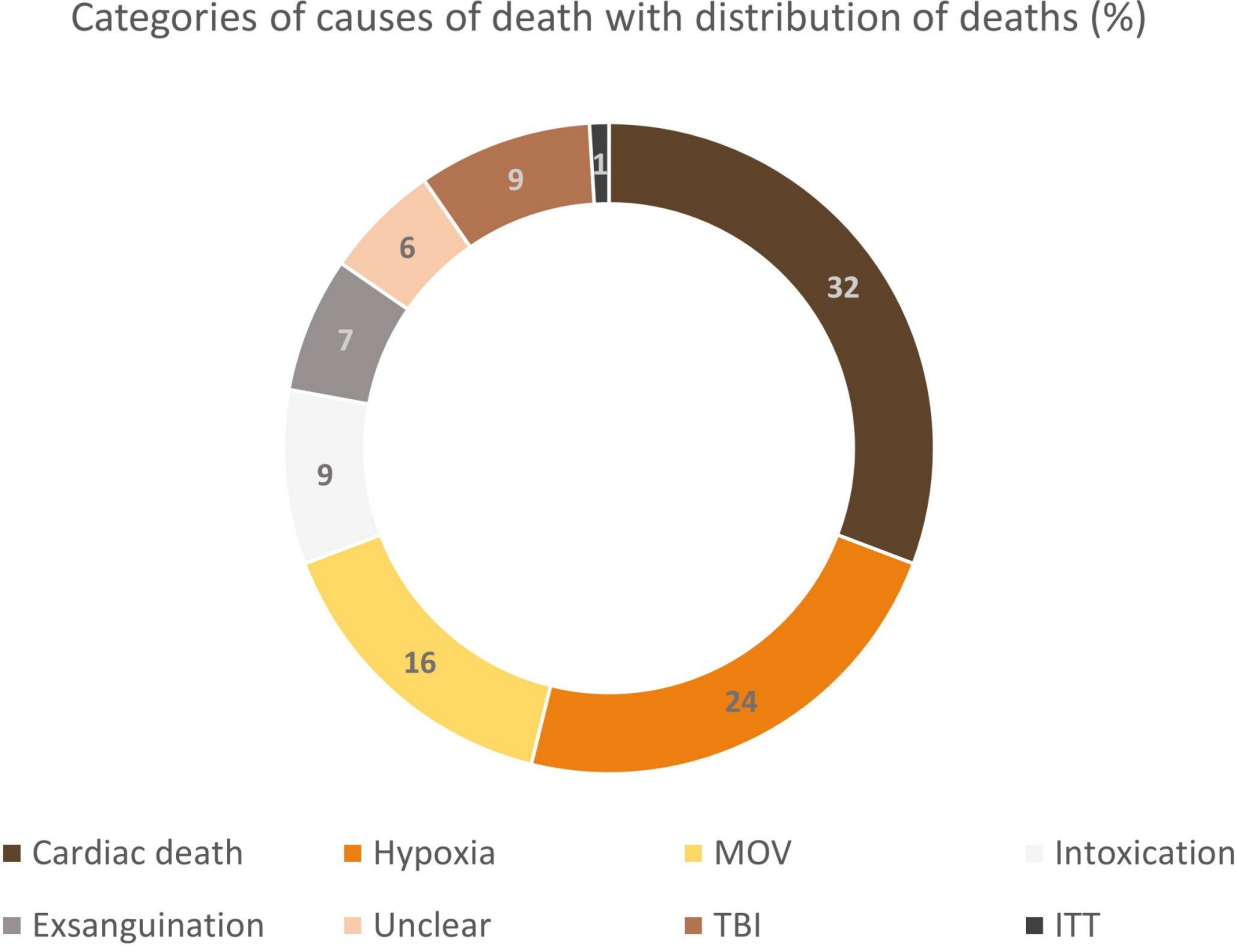
Distribution of autoptic causes of death, represented by categories: cardiac deaths (*n* = 59), hypoxia (*n* = 45), MOV (*n* = 29), intoxication (*n* = 16), exsanguination (*n* = 13), unclear (*n* = 11), TBI (*n* = 9), ITT (*n* = 1). Please note, that the colors used to represent the different groups in this graph do not correspond to the color of the respective cerebrospinal fluid (CSF) samples

The 183 cases included in this study comprised 73 women and 110 men. The age of the deceased ranged from 0 years (y) to 96 y, with a mean age at death of 62.5 y and a median of 65 y. The cohort included one intrauterine fetal death and two infants, aged 5 months and 6.5 months, respectively. The PMI was defined as the time between the assumed time of death, as noted on the death certificate, and the time of CSF sampling during autopsy. PMI values ranged from a minimum of 6 hours (h) to a maximum of 466 h, with a mean value of 129 h and a median of 128 h. Without excluding any specific causes of death, a high degree of variability was expected. Most deaths were classified as cardiac-related, totaling 59 cases (≈ 32%). Hypoxic deaths accounted for 45 deaths (≈ 24%). Deaths from MOV totaled 29 cases (≈ 16%), while deaths due to intoxication and exsanguination amounted to 16 (≈ 9%) and 13 cases (≈ 7%), respectively. In 11 cases (≈ 6%), the final cause of death could not be determined and were therefore categorized as undeterminable by autopsy. Only 9 cases (≈ 5%) resulted from TBI, and ITT was underrepresented with only one case (≈ 1%). The exact distribution is shown in Fig. 1.

Overall, we subjectively observed a high variability in the coloration of postmortem CSF, with no uniform appearance. In this qualitative evaluation of the CSF supernatants, lighter colors predominated, with 70 samples exhibiting xanthocromic / yellowish hues (≈ 38%) and 34 samples showing rose-colored tones (≈ 18%). A total of 28 samples (≈ 15%) were completely colorless, and among these, 23 samples were entirely clear, without any cloudiness or turbidity. This trend was consistent across the entire study, with most samples presenting a clear appearance.

**Fig. 2:**
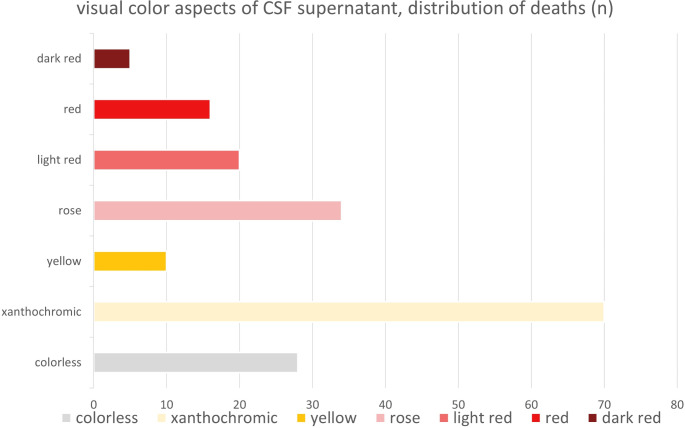
Distribution of postmortem cerebrospinal fluid (CSF) colors based on subjective qualitative analysis of supernatants, listed in descending order of frequency: yellowish / xanthochromic (*n* = 70), rose (*n* = 34), colorless (*n* = 28), light red (*n* = 20), red (*n* = 16), yellow (*n* = 10), dark red (*n* = 5)

Cases with more intense coloration were uncommon. Only 10 specimens (≈ 5%) displayed a strong yellow color, including cases associated with hypoxic deaths, MOV, cardiac deaths and intoxication. While 20 samples (≈ 11%) were categorized as ‘light red’, only 16 samples (≈ 9%) were classified as ‘red’. Additionally, in 5 cases (≈ 3%), the CSF samples exhibited an intense dark red color. These darker colors were generally accompanied by a cloudy and turbid appearance. The distribution of CSF supernatant coloration is illustrated in Fig. 2.

**Fig. 3:**
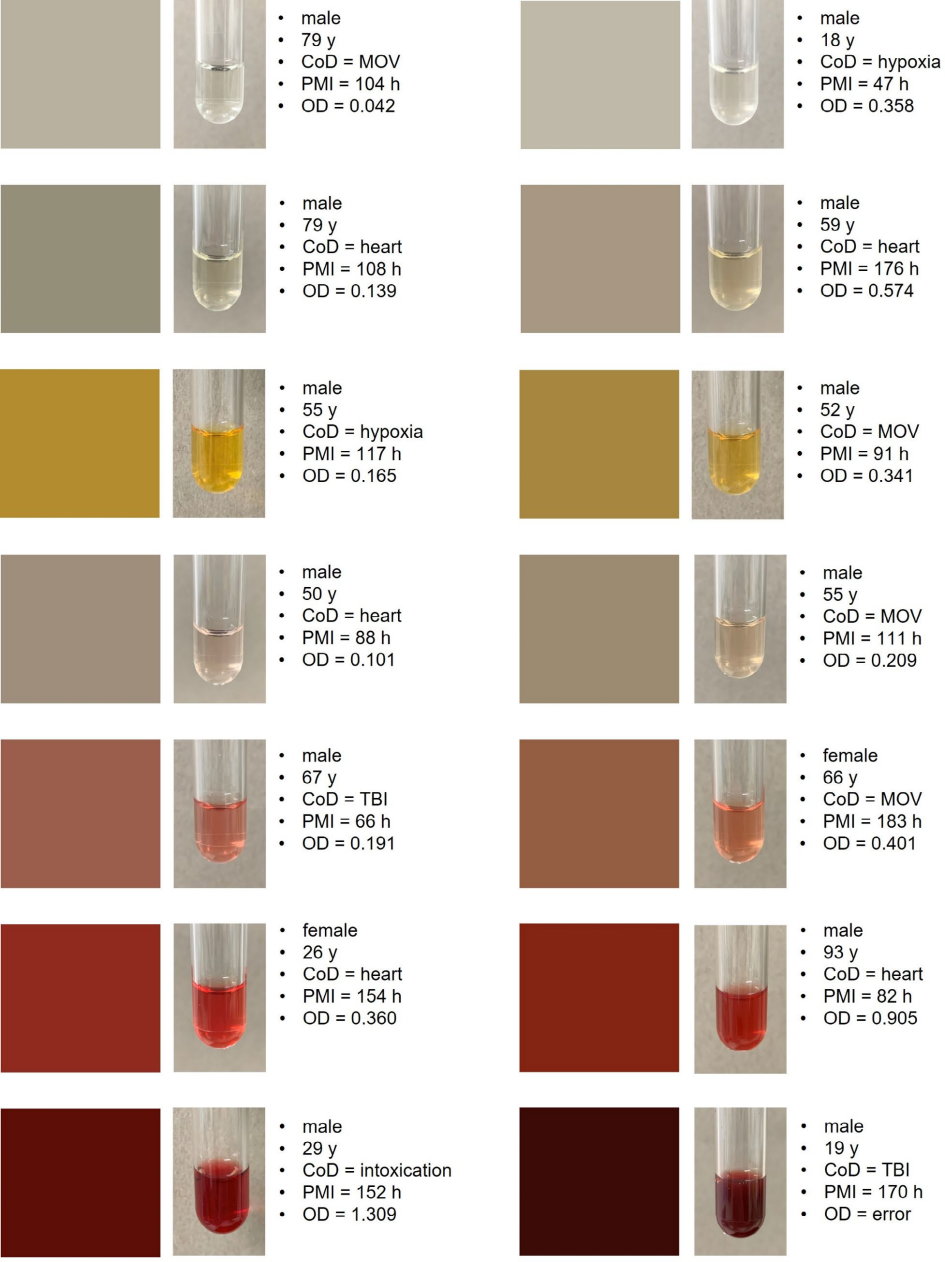
Fourteen representative cases from the cohort, with two cases assigned to each color group for visualization purposes. The left column displays CSF samples with a subjectively clear supernatant, while the right column shows samples associated with a cloudy appearance

No significant correlation was found between the CoD and the color of the CSF supernatant (*r* = 0.037) (Table 1). Two of the cases with dark red CSF were associated with TBI. Both victims died in traffic accidents involving skull fractures. Another case with dark red CSF sample showed signs of putrefaction, but sufficient CSF could still be preserved for analysis. The autopsy revealed the CoD as bolus asphyxia. The remaining two cases with dark red CSF involved deaths due to intoxication and septic MOV (Fig. 3). Red coloration of the CSF was observed multiple times in cases categorized under cardiac death, hypoxia, MOV and unclear causes of death. Only one case in this category was attributed to TBI (a fatal gunshot wound to the head) and intoxication. Intense yellow coloration, on the other hand, was present in three cases of non-traumatic brain mass hemorrhage but was also observed in cases of MOV, hypoxia, cardiac death, and one lethal intoxication. The other color groups of CSF samples were represented across almost all categories.

Furthermore, no statistically significant correlation was observed between CSF color / OD and the sex or age of the deceased (*r* = -0.012 to -0.069).

We found a highly significant correlation between the CSF color - specifically OD - and PMI (adjusted Spearman correlation p(adj) < 0.001) (Table 1). Colorless CSF supernatants were primarily observed in cases with shorter PMIs, whereas xanthrochromic staining was present in cases with both shorter and longer PMIs. The highest PMI of 466 h was recorded in a 78-year-old man who died of acute heart failure combined with hypothermia. His CSF supernatant exhibited a xanthochromic color. In contrast, the lowest PMI of only 6 h in a death, recorded in a case of death from stabbing and exsanguination, showed colorless CSF. In another case involving fatal hemorrhage after stab wounds with a PMI of 17 h, the CSF supernatant appeared light red. Similarly, a third case with the same cause of death but a PMI of 21 h showed xanthochromic CSF. Intense yellow staining of the CSF supernatant was particularly noticeable in cases with a comparatively short PMI.

The degree of turbidity was easy to assess in cases with no or only slight coloring of the CSF supernatants. Differences were clearly visible among the groups of colorless, xanthochromic and rose-colored CSF samples. However, as the intensity of color increased, particularly with the transition to dark red CSF, turbidity could no longer be adequately assessed.

**Fig. 4:**
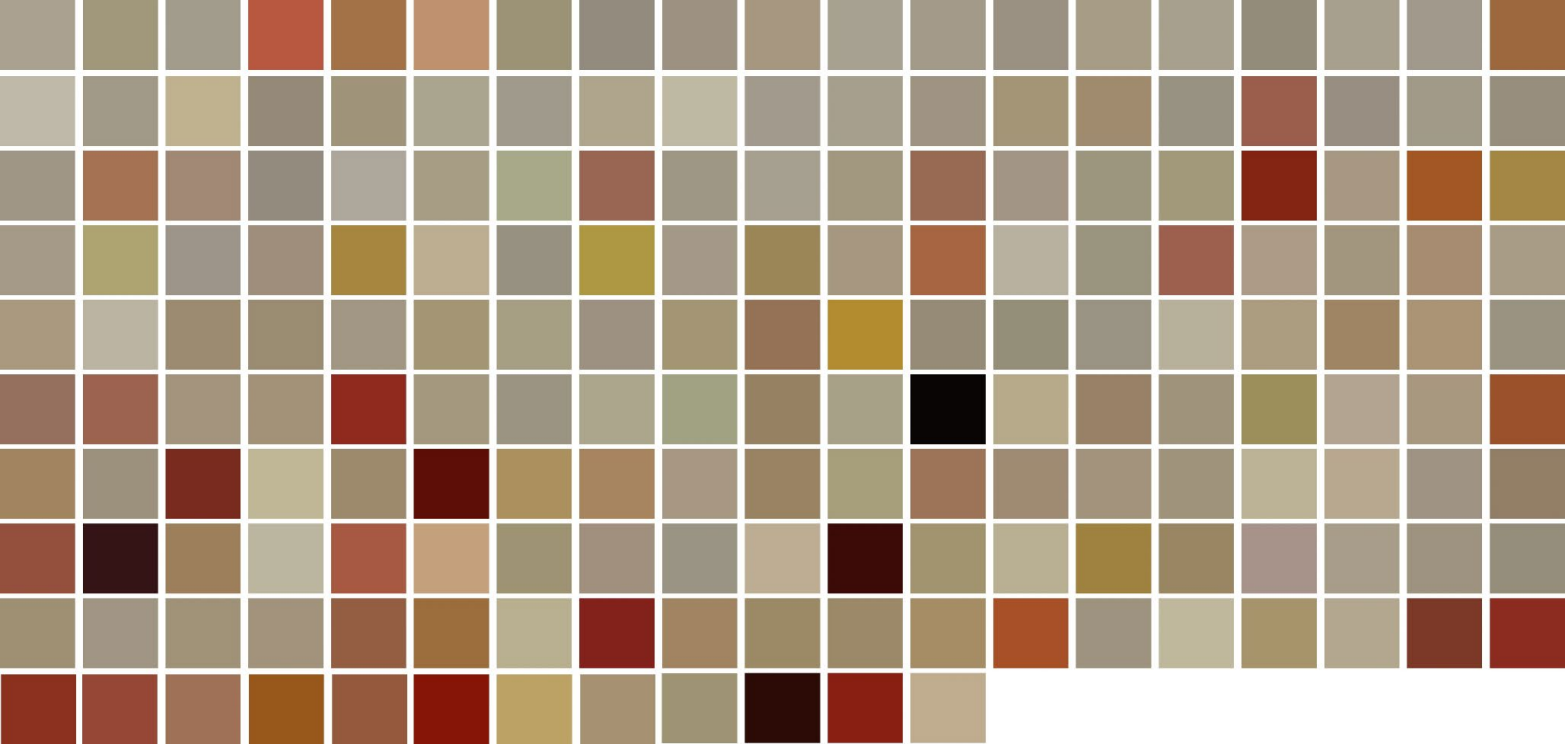
183 color tiles created for each case in the cohort using individual color codes generated with Fiji software, based on measurements from original CSF samples. The tiles are arranged to increasing postmortem interval for visualization and comparative purposes

By analyzing the RGB values of the images in ’.jpg‘ format, we were able to define an individual color code of each CSF sample and create a corresponding color tile for each case. This allowed us to visualize the overall impression of the samples in direct comparison (Fig. 4). Unfortunately, statistical comparison of the RGB with PMI was not feasible due to the RGB model’s composition of three distinct numerical values. Consequently, for statistical analysis, we employed OD and color instead of the RGB values.

**Table 1:** Spearman’s correlation matrix (p) adjusted using the Benjamini-Hochberg correction p(ad). PMI, postmortem interval; OD, optical density; CoD, individual causes of death

	Testing for correlation		
	Correlation Coefficient *r*	*p* (Spearman)	*p*(adj)
**Color vs. Age**	-0.069	0.357	0.714
**Color vs. Sex**	-0.012	0.876	0.876
**Color vs. PMI**	0.392	< 0.001 ***	< 0.001 ***
**Color vs. CoD**	0.037	0.620	0.827
**Color vs. OD**	0.602	< 0.001 ***	< 0.001 ***
**OD vs. PMI**	0.415	< 0.001 ***	< 0.001 ***

In the measurements of CSF optical density, the values for the investigated samples ranged from a minimum of 0.036 and a maximum of 58.8, with a mean value of 0.593 and a median of 0.188. Due to technical errors, it was not possible to determine values for three cases despite repeated measurements. All three of these cases involved dark red samples (two cases of lethal TBI and one case of bolus death). Overall, higher OD values were associated with more intense, darker coloring of the CSF supernatant, showing a significant correlation (p(adj) < 0.001) (Table 1). Colorless to light-colored CSF samples generally had lower values, while CSF samples with a cloudy appearance tended to show higher OD measurements (Fig. 3).

## Discussion

With a few exceptions, the biochemical examination of postmortem CSF is not part of routine diagnostics in legal medicine [2]. In the clinical setting, however, the analysis of CSF is an obligatory diagnostic tool [16]. Previous have already demonstrated the equally high potential of CSF examination for postmortem issues [7, 12, 14, 17–20]. The assessment of CSF is not only important in clinical contexts but also represents an easily accessible medium with potentially diverse informative value in postmortem analysis. For example, CSF examinations can provide insights into neuropathological conditions and can also help determine the cause and time of death [2, 21–23]. Although postmortem CSF is attracting increasing interest, its examination sometimes requires additional and complex pre-measurement procedures. In our study, we focused on the visual appearance of CSF samples as a baseline indicator of the fluid’s potential usefulness, particularly regarding its color. We detected differences in visual appearance that correlated with PMI. Colorless CSF supernatants with lower OD values were commonly observed in cases with shorter PMI, while CSF supernatants with more intense, darker coloring and higher OD values were associated with longer PMI. The presence of pigments, such as oxyhemoglobin or bilirubin, appears to alter CSF coloration depending on PMI. This could potentially serve as a diagnostic tool in the forensic methodology spectrum for PMI determination in the future.

We used puncture of the spinal canal through the foramen magnum as a simple way of preserving our CSF samples during postmortem examination. This technique was used for all 183 samples analyzed. Alternative common procedures for CSF sampling such as lumbar puncture (LP) or aspiration of the cerebral ventricles [24] were not applied. The potential influence of the sampling technique on the visual color aspect of the CSF can therefore not be assessed.

In the study, some causes of death, such as traumatic causes, were underrepresented in the cohort. Consequently, the potential effects of trauma (accompanied by fractures) on CSF staining are more challenging to classify in comparison to cardiac deaths, which comprised the majority of cases in our study and in all subsequent autopsy cohorts. However, a significant blood admixture in the CSF was not observed in the TBI group, the single ITT case, or in previous studies conducted by our group [2]. Most of the cases investigated were male and older. No significant correlation between age or sex and the color or OD of postmortem CSF was established. In all three pediatric cases, the CSF appeared rose-colored. Intense staining of postmortem CSF was observed in both younger and older individuals. One aim of this descriptive study was to compile a representative cohort of autopsy samples with minimal exclusion criteria.

As additional information, we collected only data on sex, age, PMI, and CoD for the deceased. The influence of potential other factors (such as medication and drug abuse, time of agony, pre-existing diseases, body temperature and external factors related to the storage conditions of the bodies) on CSF coloration was not investigated. However, it is known that medication can influence the central nervous system at a cellular level [25, 26], and potentially affect the CSF as well. Certain medications can, for example, lead to increased protein levels in the CSF, which may impact its appearance in terms of color and, especially, turbidity [27]. Therefore, collecting more comprehensive data in future comparative studies would be beneficial.

Only one of the examined bodies showed early signs of putrefaction. In such cases, determining the exact PMI can be challenging and remains one of the greatest difficulties in legal medicine [28]. Consequently, a bias in the results related to PMI determination cannot be ruled out.

In the given study, postmortem CSF exhibited a highly diverse range of appearances rather than a uniform one. Our analysis identified various color spectra, which we categorized into polychromatic categories. Fine graduations in color shades were required, and the classification into these groups was somewhat subjective, although it was conducted according to the four-eyes principle. Due to practical constraints, no second or third rater was included in the study design. Alternative group allocations could be considered, and efforts should focus on maximizing reliability. Contrary to a pessimistic view that cadaveric CSF would be bloody or at least hemolytic in almost all cases, we identified 28 cases with completely colorless postmortem CSF. In these samples, the visual appearance was consistent with the physiological, crystal-clear CSF seen in living patients.

In the clinical setting, xanthochromia is often discussed in relation to the diagnosis of subarachnoid hemorrhage (SAH) based on the breakdown products of blood cells [27, 29]. In 2003, Seehusen et al. described the visual analysis of CSF and CSF supernatant, associating different color shades with various clinical diagnoses [27]. In contrast to their classification, our study identified finer gradations in the color qualities of CSF samples, leading to the grouping presented in our findings. We were unable to reproduce all of the CSF staining descriptions, such as the brownish color of CSF supernatants. It is important to note these observations were based on samples from living patients, not postmortem ones. Yellow coloring of CSF is also associated with liver failure and generalized, severe hyperbilirubinemia, and is sometimes seen in newborns due to elevated bilirubin levels [27]. Contrary to the expectation that yellow CSF would be common in liver failure, we detected this coloring in only one case of MOV with liver cirrhosis. In three other liver failure cases, the postmortem CSF had different colors. Regarding TBI, only two cases showed light yellow CSF, while cases with cerebral mass hemorrhage sometimes exhibited yellow CSF. Thus, postmortem CSF analysis was partially consistent with clinical observations. However, rose to red discolorations of postmortem CSF, which were not reported by Seehusen et al., were observed with similar frequency in cases of intracranial bleeding.

In general, we observed a wide range of CSF colors in our study. There was no correlation between CSF color or OD and CoD. Consequently, we found that nearly all color spectra could be summarized across different groups. Particularly with regard to TBI, red, blood-tinged CSF was not consistently detectable in our postmortem trauma samples. Thus, it is not possible to infer an underlying TBI solely based on reddish postmortem CSF according to our observations. Similarly, yellowish to yellow CSF did not reliably indicate intracranial bleeding or liver failure.

Our results indicated that OD increased with the intensity of CSF color. This was also true for the subjectively assessed turbidity of our CSF samples. However, we detected equally turbid but colorless CSF, which still showed low OD values. Further investigations using objective measurement methods, such as advanced photometry techniques, could validate our observations.

In summary, we focused on the visual appearance of CSF samples routinely collected during autopsy. Contrary to the pessimistic view that cadaveric CSF is predominantly bloody or at least hemolytic, many samples clear and transparent, without blood contamination. We found a highly significant correlation between CSF color / OD and the PMI. This finding is crucial, as determining the PMI remains a challenging aspect of postmortem examinations [28]. This study suggests a promising enhancement to conventional PMI estimation methods by introducing a rapid and accessible approach utilizing CSF color and OD.

### Limitations

Our observations are ultimately based on the impressions gained during CSF inspection, which introduces potential subjectivity, as well as additional objective measurements of OD. It is important to note that despite careful sampling techniques, minimal blood admixture during CSF collection - and its influence on the staining of CSF supernatants – could not be definitively ruled out. Blood admixtures may have also occurred iatrogenically during autopsy, though proper centrifugation of the samples have minimized this effect. For samples in the ‘dark red’ group, we were unable to determine OD even after multiple measurements. The causes or cut-off values for CSF samples with intensive dark staining remain undetermined. Creating color tiles for each case aimed to provide visual comparability and illustrate the diversity of postmortem CSF colors. However, using a uniform grey background [15] for photographic documentation introduced a bias, a affecting the resulting color codes in by influencing the RGB component evaluation. As a result, color tiles of clear, colorless CSF samples appear greyish rather than ‘white’. This is a systematic error affecting all cases included. Further investigations could benefit from comparing CSF before and after centrifugation to clarify the influence of cellular components on the initial visible quality of CSF during and after sampling. Our study focused on illustration the immense diversity of postmortem CSF. Additional biochemical analyses were not performed and should be linked in future studies. The examination of other body fluids (e.g. vitreous humour) should be also included and will be addressed in subsequent investigations.

### Key points

1. Postmortem cerebrospinal fluid exhibits a diverse range of appearances, with varying colors, and often shows a clear appearance similar to physiological clinical samples of cerebrospinal fluid.
2. There is a highly significant correlation between the color / optical density of the cerebrospinal fluid and the postmortem interval.
3. No correlation has been found between the color / optical density of the cerebrospinal fluid and the causes of death.
4. Optical density and turbidity increase with the intensity of color of the cerebrospinal fluid.
